# Anthrax: Transmission, Pathogenesis, Prevention and Treatment

**DOI:** 10.3390/toxins17020056

**Published:** 2025-01-24

**Authors:** Nitika Sangwan, Aakriti Gangwal, Preksha Jain, Chokey Langtso, Shruti Srivastava, Uma Dhawan, Renu Baweja, Yogendra Singh

**Affiliations:** 1 Department of Biomedical Science, Bhaskaracharya College of Applied Sciences, University of Delhi, Delhi 110075, India; 2 Department of Medicine, Division of Infectious Diseases and Geographic Medicine, Stanford University, Stanford, CA 94305, USA; 3 Dr. B.R. Ambedkar Center for Biomedical Research, University of Delhi, Delhi 110007, India; 4 Department of Biochemistry, Shivaji College, University of Delhi, Delhi 110027, India

**Keywords:** sporulation, germination, bacterial toxin, pathogenesis, anthrax vaccine

## Abstract

*Bacillus anthracis* is a deadly pathogen that under unfavourable conditions forms highly resistant spores which enable them to survive for a long period of time. Spores of *B. anthracis* are transmitted through the contaminated soil or animal products and enter to the host through the skin, lungs or oral route and can cause cutaneous, injection, inhalation and gastrointestinal anthrax, respectively. The disease is caused by the toxin which is produced by them once they germinate within the host cell. Anthrax toxin is the major virulence factor which has the ability to kill the host cell. The role of protein kinases and phosphatases of *B. anthracis* in toxin production and other virulence related properties have also been reported. There are two vaccines, BioThrax and CYFENDUS^TM^, which are approved by the FDA-USA to prevent anthrax disease. Recently, anthrax toxin has also been shown to be a potential candidate for cancer therapeutics. Through present review, we aim to provide insights into sporulation, transmission and pathogenesis of *B. anthracis* as well as the current state of its prevention, treatment, vaccines and possible therapeutic uses in cancer.

## 1. Introduction

**Figure 1:**
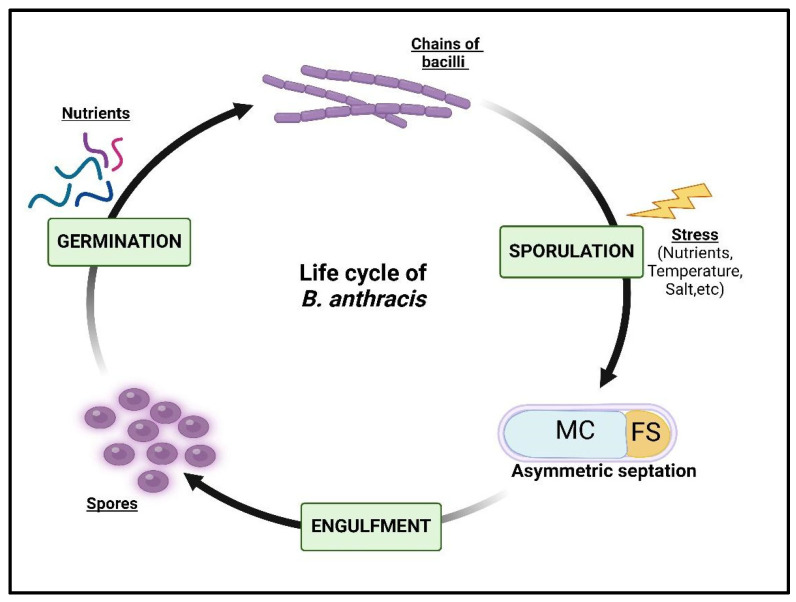
Life cycle of *Bacillus anthracis*. MC: Mother Cell; FS: Forespore.

The life cycle of *Bacillus anthracis* is centered around two distinct phases, namely sporulation and germination, which are well-explored processes. In brief, sporulation is a multi-step process involving asymmetric septation of the vegetative bacillus resulting in the formation of two compartments, the mother cell and the forespore. Following this, the forespore remains engulfed by the mother cell and once spores mature, they are released outside the environment (Figure 1). These spores, being highly resistant, can survive and remain dormant for years until favourable conditions resume [1,2]. Once transmitted, these deadly spores can enter the host and are responsible for causing anthrax. The pathogenesis and survival of *B. anthracis* is a well-studied phenomenon. Lately, it has been reported that protein phosphorylation has a significant role in the pathogenesis of *B. anthracis* by modulating various cellular processes essential for its survival, virulence and ability to evade host immune responses [3,4]. Targeting protein phosphorylation pathways in *B. anthracis* offers a promising approach for developing new drugs against this deadly pathogen. Although there are advancements in the treatment of anthrax, the effectiveness of existing antibiotics, small-molecule inhibitors and vaccines depends upon the timing of their administration. The gaps pertaining to the spore-specific therapies, toxin-mediated damage and late-stage interventions persist and continued research into anthrax pathogenesis and host–pathogen interactions is critical in ensuring better preparedness and more effective management of anthrax.

## 2. Sporulation and Germination of *Bacillus anthracis* Spores

The formation of spores is the crucial aspect of the life cycle of *B. anthracis*, which enables its survival in adverse environmental conditions. Their spores can survive and remain dormant under extreme environmental stresses including nutrient deprivation, heat, UV radiation, acid, alkali and other chemical agents [1] and are also easily transmitted through air, water or soil. Once transmitted, these spores can come into contact with their potential host (human or animal), wherein they germinate and eventually cause infection. Importantly, *B. anthracis* spores are proven to be a potential bioweapon, which is of constant concern worldwide [2,5].

**Figure 2:**
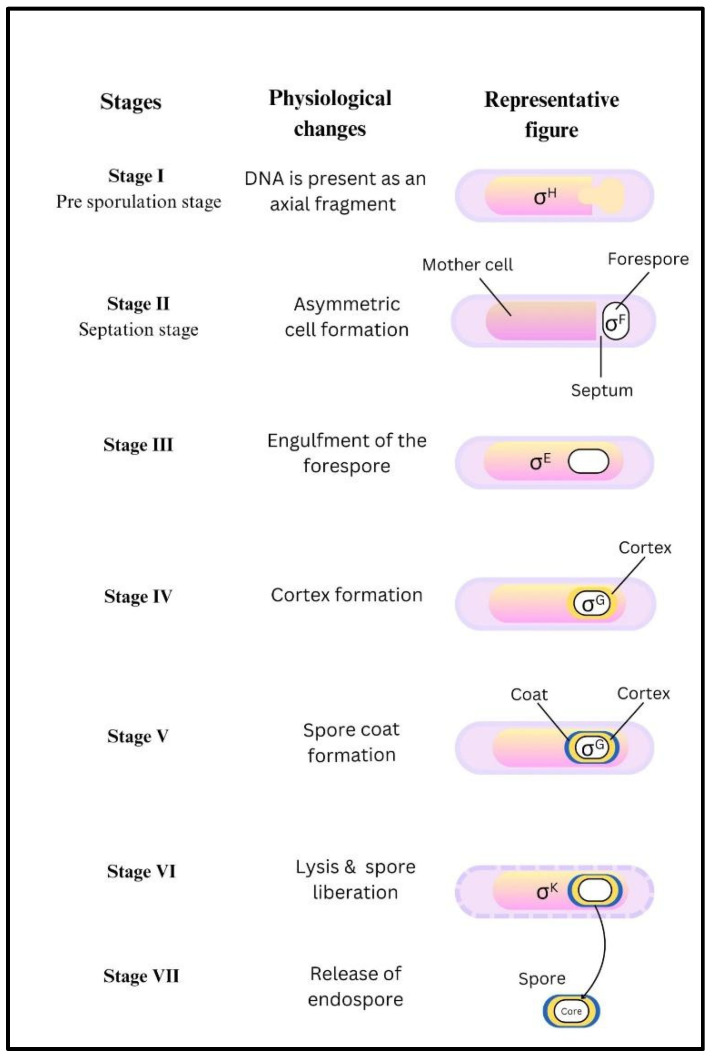
Stages of sporulation in *Bacillus anthracis*.

The formation of spores through sporulation and the conversion of spores to vegetative cells through germination are the key steps in the life cycle of *B. anthracis* [6]. The spore formation takes place in a number of stages as follows: stage I is the initiation stage, wherein DNA is present as an axial filament; this is followed by the formation of septum and asymmetric cell formation of the mother cell and forespore in stage II. The forespore is engulfed by the mother cell in stage III, followed by the formation of cortex in stage IV. The synthesis of spore coat proteins, dipicolinic acid (DPA) and uptake of calcium in the spore core commences in stage V. In stage VI, spores become mature and retractile and are finally released from the mother cell in stage VII (Figure 2).

The phenomenon of sporulation is under the tight control of five sigma factors which regulate the cells at both the transcriptional and physiological level [7]. These sigma factors, which are sequentially expressed, temporally regulate the expression of mRNAs to ensure the compartment-specific expression of genes required for spore formation [8]. Firstly, σ^H^ is expressed towards the end of the exponential and beginning of the stationary phase of the growth to initiate the sporulation process. It also directs the expression of the genes needed for the activation of subsequent sigma factors including σ^F^ and σ^E^, which are early forespore-specific and early mother cell-specific sigma factors. σ^F^ not only helps in forespore development but also directs the formation of the spore core. σ^E^ controls the expression of mRNAs required for the mother cell engulfment of the forespore [9]. Following this is spore maturation, wherein the formation of spore cortex and coat proteins takes place under the control of σ^G^, and it has been reported that the “Fin” protein is required for efficient switching from σ^F^ to σ^G^ [10]. Last, there is an expression of σ^K^, which finalizes the coat formation, and ultimately the mature spores from the mother cell. In addition to its role in sporulation, σ^H^ is also shown to be a key independent player for regulating the expression of anthrax toxin [11].

Interestingly, there are many other proteins which affect the formation of spores. One such protein is spoVG. The sporulation of the Δ*spoVG* mutant of *B. anthracis* vaccine strain is affected and blocked after stage I, leading to the absence of compartmentalization between MC and FS due to defects in the formation of the asymmetric septum [12]. Similarly, the Spo0E family of proteins is also shown to negatively regulate the sporulation pathway of *B. anthracis* by dephosphorylating the Spo0A response regulator, a master response regulator of initiation of sporulation [13]. Furthermore, multiple genes have been shown to affect the sporulation and germination of *B. anthracis*. Liszewski Zilla M et al. (2015) studied the role of LCP (LytR-CpsA-Psr) enzymes in growth and sporulation of *B. anthracis* [14]. LCP enzymes are universal enzymes which transfer undecaprenyl-phosphate polymers to peptidoglycan in Gram-positive bacteria and in *B. anthracis*; they are six in numbers namely LcpB1-4, LcpC and LcpD. The study showed the significant role of LcpB1, LcpB2 and LcpB4 in the sporulation of *B. anthracis* with a substantial decrease in sporulation efficiency in strains lacking these three enzymes. In another study, the role of the McsB and ClpC proteins of the clpC operon was demonstrated in the sporulation and germination of *B. anthracis*. Reduced sporulation efficiency by 40% and 60%, respectively, was observed in *ΔclpC* and *ΔmcsB* even after 144 h of the growth under suitable growth conditions. The spores formed were defective with respect to their coat layer formation, resulting in a drastic reduction of their germination with efficiency of 40% in case of *ΔmcsB* and 10% in *ΔclpC* [15]. PrpN, a serine/threonine protein phosphatase of *B. anthracis,* was also reported to play an indispensable role in its sporulation and germination. Sporulation was delayed in the *ΔprpN* strain of *B. anthracis*, with a sporulation efficiency of only 0.01% as compared to the wild-type strain. These spores were also structurally abnormal with the missing spore layers [16].

**Figure 3:**
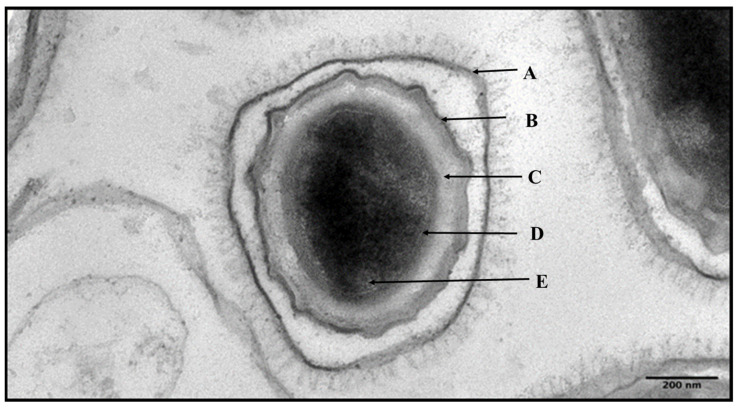
Transmission electron micrographs of *Bacillus anthracis* spores formed under normal growth conditions (Adapted and modified from [28]). Arrows represent the different layers of a mature spore: exosporium (A), spore coat (B), cortex (C), core wall (D) and core (E).

The spores of *bacillus* species may remain quiescent for many years and can resist a wide range of stresses [1,2,17] due to their unique protein composition and structural and physical features (Figure 3). Proteomic studies of the spores have revealed the presence of 500–800 numbers of proteins depending upon the strain and stage (dormant or germinating spores) of the bacillus and also the experimental methods used for the analysis [18,19,20]. Structurally, the spore cytoplasm (core) is poorly hydrated and contains high concentrations of small acid-soluble proteins (SASPs). These SASPs, along with high levels of calcium-dipicolinic acid (Ca-DPA) and other ions, are responsible for their resistance to heat and ultraviolet radiation [21,22]. The core also comprises DnaK and GroE, heat shock proteins, which become activated during stress and aid in protein folding during germination. The RecA core protein helps to repair DNA damages that occur during spore dormancy. The dehydrated state of the core is also governed by the core membrane and a surrounding peptidoglycan layer, which restrict the entry and exit of water and other solutes [2]. These dehydrated conditions help to maintain the spore dormancy. Further, the cortex hydrolases such as SleB and CwlJ are important for the degradation of the cortex during spore germination [23]. The cortex is surrounded by a coat which contains several proteins and has been shown to confer resistance to several degradative enzymes like proteases and lipases and other sporicidal substances [24]. The coat layer also serves as a barrier by protecting the spore from toxic molecules [22]. For example, cotα, an outer spore coat protein of *B. anthracis*, provides resistance to phenol (10%), chloroform (10%) and hypochlorite (4%) [25]. However, it is not impermeable, as it provides access to germinants to the receptors present on the inner forespore membrane and hence has a role in germination [26]. The coat layer is surrounded by the exosporium, which is made up of a basal layer and an external hair-like structure of glycoprotein called BclA [2,8,22]. This is the outermost layer of the spore and may play a major role in spore adherence and its aggregation by affecting their electrostatic properties, which determines their dispersal and infectivity [22]. Besides the main structural components described above, the spore core is also surrounded by two membranes. The inner spore membrane surrounds the spore core and is believed to be the house of germinant receptors responsible for sensing the number of small molecules that act as triggering agents of germination [21]. The outer spore membrane is thought to contain the cortical degradative enzyme SleB and its stabilizing protein YpeB, but their locations are not yet certain and it has been suggested that these enzymes are present in the spore coats [27].

**Figure 4:**
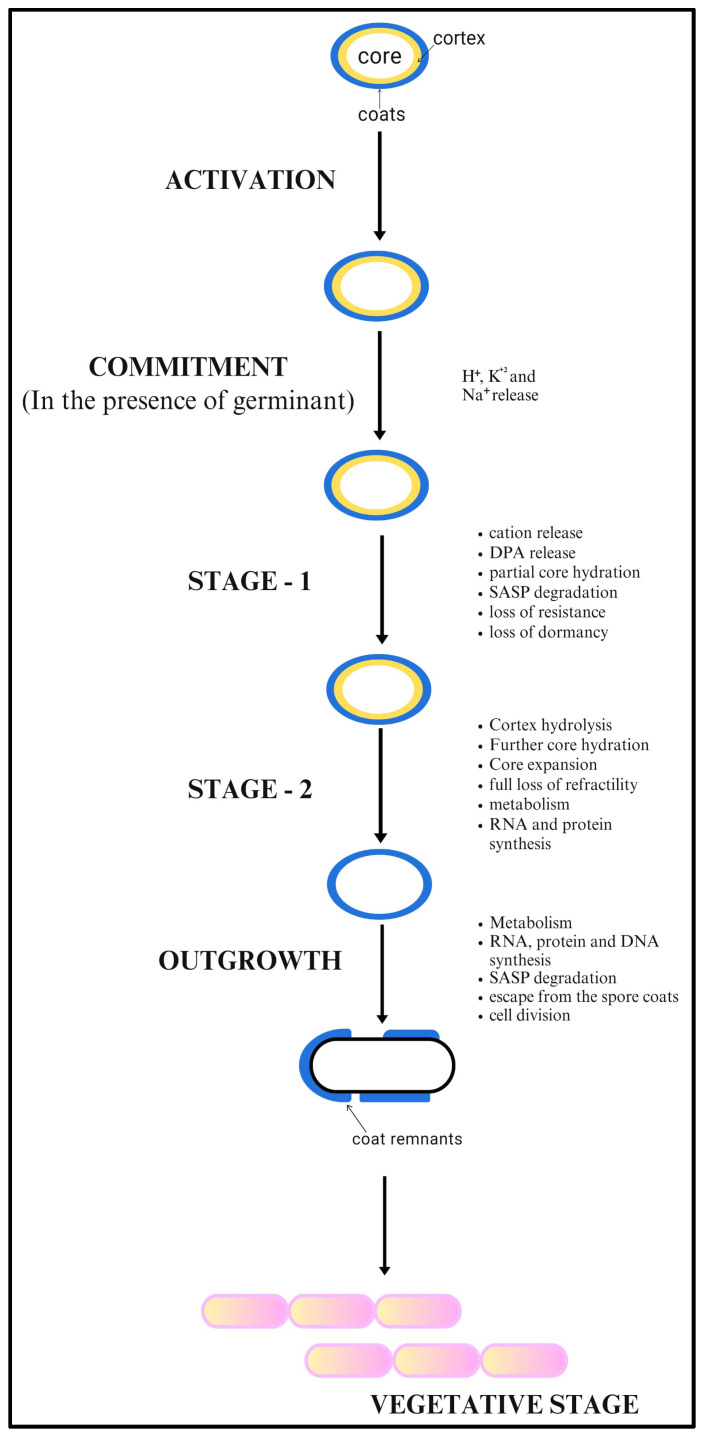
Stages of germination and outgrowth in *Bacillus anthracis*.

Spores can remain in a dormant state for years. Under suitable environmental conditions and in the presence of germinants, they lose their dormancy and germinate. These germinants are small biomolecules such as sugars, purine nucleotides, amino acids or their combinations [29]. As soon as spores are mixed with germinants, germination begins due to the activation of germinant receptors [21,30,31]. In *B. anthracis*, there are five functional germinant receptors encoded by the tricistronic operon. Four of these, namely *gerH*, *gerK*, *gerL* and *gerH*, are located on the chromosomal DNA, and the fifth one, namely *gerX*, is located on plasmid pXO1. Further, the access of a germinant to its receptor is also reported to be facilitated by specific proteins. GerP protein is shown to be required for rapid germination of *B. anthracis* spores, probably by increasing the access of germinants to their receptors [32]. Once receptors recognize their specific germinants, a series of events is initiated. Firstly, the spore core releases monovalent ions, including K^+^, Na^+^ and H^+^, which results in the increase in the pH of the spore core from slightly acidic to basic [33]. The core becomes rehydrated by releasing Ca-DPA and its associated divalent ions. The release of Ca-DPA is thought to be governed by the SpoVA protein associated-channels of the spore’s inner membrane [34]. This completes the stage I of the spore germination. During stage II, the peptidoglycan (PG) layer of the spore cortex becomes degraded by cortex-lytic-enzymes (CLEs) via the modification of its muramic acid-δ-lactum residues. The two major CLEs of *Bacillus* species are CwlJ and SleB, which, when acting together or alone, hydrolyse the spore cortex PG layer [35,36]. The removal of the PG layer causes further uptake of water, which enhances core rehydration resulting in the spore core with 80% wet weight as water [37]. The increase in water content helps in enhancing protein mobility in the core and degradation of SASPs to release amino acids. These SASPs constitute around 10% of the spore core proteins, in which they associate with DNA (α/β) and protect it from heat and oxidizing agents [37,38]. The degradation of SASPs is essential to provide access for proteins to core DNA for transcription during the spore outgrowth. The increase in water content of the spore core helps to activate the metabolic enzymes of the spore core, resulting in synthesis of macromolecules required for vegetative cell formation. Germination is followed by the spore outgrowth, in which spores develop into vegetative cells which are capable of normal growth and division (Figure 4) [33,36,38].

## 3. Transmission of *Bacillus anthracis* Spores

In order to effectively manage and control anthrax outbreaks, it is important to understand the transmission of *B. anthracis* spores [39]. Being a potential bioweapon, knowledge of its transmission further aids in designing strategies for biodefense. The transmission of spores primarily occurs via contaminated soil, animal products or carcasses of infected animals through various routes such as cutaneous contact, ingestion or inhalation resulting in cutaneous, gastrointestinal or inhalation anthrax, respectively [39].

### 3.1. Cutaneous Anthrax

In cutaneous anthrax, spores enter through skin abrasions, and therefore, anthrax is generally associated with occupational contact. Butchers, wool processors and animal handlers are at higher risk [40]. It can also be transmitted by non-biting flies which can deposit spores or bacilli on abrasions or by biting flies which can transfer it directly to blood through open wounds. The spores could be picked up from the carcass when they are present on their body and also in their crops or intestines [41]. Cutaneous anthrax accounts for more than 90% of cases of anthrax infection. Its incubation period varies from 2–6 days, after which there is a development of skin lesions, which are usually painless and pruritic papules. In 2–3 days, the lesion matures, undergoes necrosis and finally forms a characteristic black dead tissue surrounded by edema [42,43]. The fatality rate is 20% without antibiotics and <1% with antibiotic treatment [44].

### 3.2. Gastrointestinal Anthrax

It mainly occurs in animals grazing on contaminated pastures and in humans by ingesting inadequately cooked contaminated food products. The incubation period of the bacilli ranges from 3–7 days, and the lesions can develop at any location throughout the gastrointestinal lining. The initial symptoms include fever, vomiting, nausea, diarrhoea and abdominal pain. With further progress in infection, severe symptoms are observed including acute–unbearable abdominal pain, bloody diarrhoea, septicemia shock and eventually death. Unlike cutaneous anthrax, its mortality rate is higher with up to 60%, if untreated and around 25% with proper treatment [42,43,45].

### 3.3. Inhalation Anthrax

With the highest rate of mortality at 45% with antibiotic and >95% without antibiotic treatment, it is the most dangerous form of anthrax [40]. It occurs due to the inhalation of pathogenic spores. Within the lungs, these spores become engulfed by the immune cells and then are transported to the lymph nodes. The toxins produced by the bacteria in the lymph node disrupt their integrity and enter the blood, causing bacteremia and the death of the host. The appearance of the symptoms is biphasic, with initial symptoms appearing 1–7 days after infection, and are mainly due to the infection in the upper respiratory tract [39]. The symptoms include fever, chills, fatigue and non-productive cough. This is followed by more severe symptoms of chest pain, breathing difficulties and high fever. Without medication, the condition worsens, resulting in septicemia, edema and fatal shock.

### 3.4. Injection Anthrax

It is the rarest form of anthrax, which is caused by the entry of *B. anthracis* spores through intravenous, subcutaneous or intramuscular injection of contaminated drugs. The infection causes severe edema at the injection site, resulting in abscess or necrotizing fasciitis [46]. To date, three outbreaks of injection anthrax have been reported among heroin users in Europe, with a mortality rate of up to 35% [47]. Besides antibiotic treatment, surgical removal of the infected region is necessary to treat the infection.

Meningitis due to *B. anthracis* infection is a rare but fatal form of anthrax. The anthrax bacilli can enter through the skin or the oral or respiratory route, spread via blood or lymph to the central nervous system after crossing the blood–brain barrier and cause meningitis [48].

## 4. Virulence Factors

Bacterial pathogens are known to have multiple virulence factors comprising proteins, carbohydrates, lipids, etc. Some bacterial toxins comprise two components, one having enzymatic activity (A) and other having the ability to bind receptors (B). Therefore, these toxins are also known as AB toxins. The receptor-binding component binds to the receptor and delivers an enzymatic moiety to the cytosol, which can cause deregulation of cellular metabolism, leading to cell death. Bacterial toxins are known to exhibit DNAse, RNAse, ADP ribosylation and other enzymatic activities. The arrangement of enzymatic component and receptor-binding component varies among various toxins. While in some bacterial protein toxins, both A and B components are present on the same polypeptide, in many toxins, these are present on different polypeptide chains. Different bipartite bacterial toxins are released by *Pseudomonas*, *Streptococcus*, *Salmonella*, *Bacillus*, etc. [49]. 

**Figure 5:**
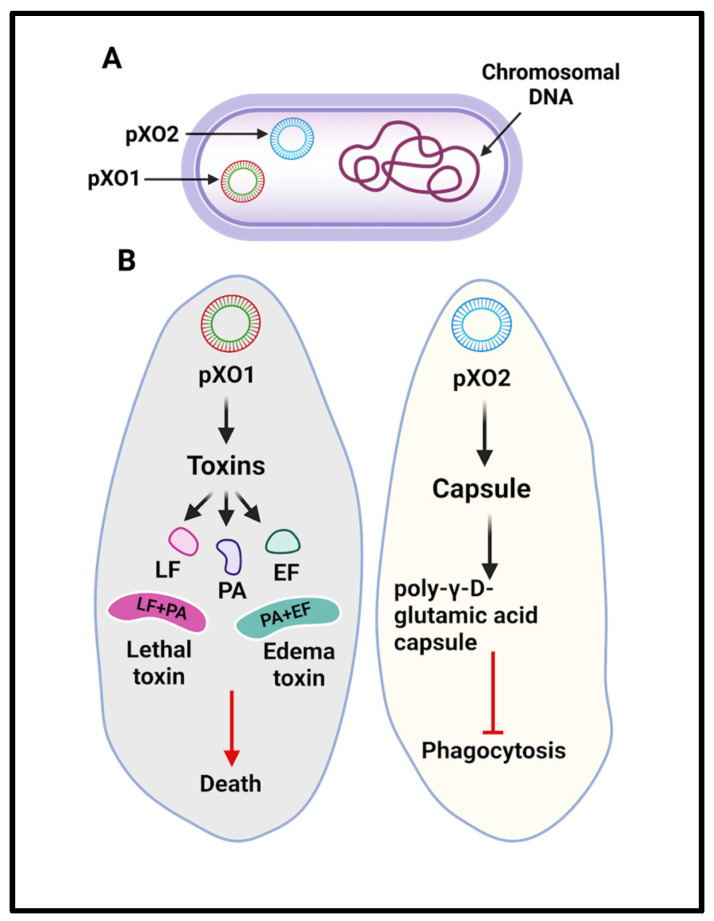
(**A**) *Bacillus anthracis* cell with pXO1 and pXO2 plasmid. (**B**) Virulence factors encoded by pXO1 and pXO2.

*B. anthracis* virulence factors comprise a tripartite toxin and an antiphagocytic polypeptide capsule. The genetic foundation of these virulence factors lies within pXO1 and pXO2 plasmids. The anthrax toxins, namely Protective Antigen (PA), Lethal Factor (LF) and Edema Factor (EF), are encoded by pXO1, while pXO2 encodes the capsule that prevents phagocytosis [50] (Figure 5).

**Figure 6:**
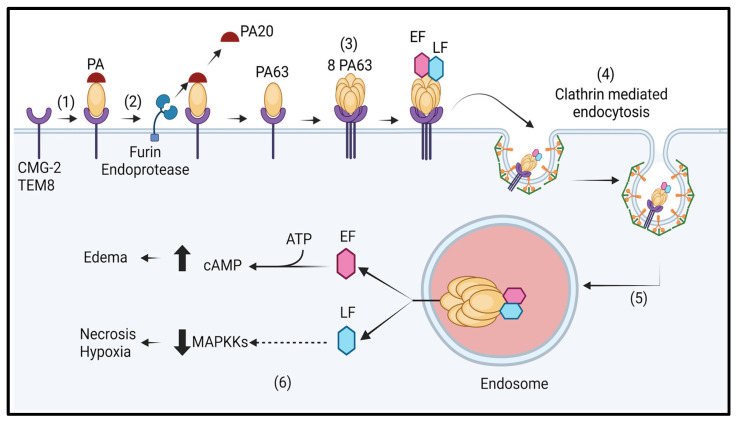
Schematic representation of mechanism of anthrax toxins action inside the host: (1) Binding of PA to CMG2. (2) Cleavage of N-terminal of PA (20 KDa) by furin-endoprotease. (3) Oligomerization of the cleaved part of PA (63 KDa) into ring-shaped octamer, which binds to LF and EF. (4) The internalization of the complex through clathrin-mediated endocytosis. (5) PA undergoes acidification inside the endosome and forms a narrow channel, leading to translocation of LF and EF into cytosol. (6) LF (Zn-dependent protease) acts on the MAPKK pathway, leading to disruption of host cellular pathways, while EF (calmodulin-dependent adenylyl cyclase) converts ATP into cAMP and disrupt cellular homeostasis leading to death of host. The up-arrow shows the increase in cAMP levels and down-arrow shows the disruption of MAPKK pathway.

These virulence factors allow the bacteria to enter into the host cell, escape the immune system and responses and induce severe systemic infections that can be fatal if untreated [50,51]. The pathogenicity of *B. anthracis* hinges on its ability to subvert the host’s immune defences and establish a systemic infection. This begins with the germination of spores in the infected host and release of two major toxins namely lethal toxin and edema toxin. The lethal toxin consists of PA protein and an enzymatic moiety, LF, whereas edema toxin contains PA protein and the EF enzymatic moiety. Individually, these three toxins are harmless. Both LF and EF require binding to PA for expression of their respective activity. PA, which is a 83 kDa-soluble protein, serves as a central component for the other two toxins (Table 1). LF is a Zn-dependent protease and cleaves the mitogen-activated protein kinase kinases (MAPKKs), leading to the inactivation of intracellular signalling cascades of the host [52,53]. This interference hampers the host’s immune response and contributes to the bacteria’s ability to evade immune surveillance [54]. EF, an adenylate cyclase act by increasing intracellular cyclic Adenosine Monophosphate (cAMP) levels, disrupting cellular homeostasis by interfering with various cellular functions, including immune cells and contribute to the characteristic edema observed in anthrax infections [55,56]. PA binds to capillary morphogenesis protein-2 (CMG2) or tumor endothelial marker-8 (TEM8) by its C-Terminal with the help of metal-ion dependent adhesion site (MIDAS) coordinating the divalent ions and facilitates entry of LF and EF into host cells. After binding, the 20 kDa sequence of PA is cleaved by the furin family of cellular proteases at the sequence RKKR. After the sterically inhibiting unit of PA20 is removed, the cleaved 63 kDa PA now forms a ring shaped octamer. The oligomerization of the PA63 takes place by refolding in the FFD (315) domain. The oligomer can bind to maximum 3 molecules of LF and EF [57]. PA-bound LF or EF are engulfed via receptor-mediated endocytosis. This is followed by the acidification of endosomes which facilitate the PA insertion into the endosome membrane, forming a channel through which the unfolded form of LF and EF can translocate to the cytosol via Brownian motion [58,59] (Figure 6).

Anthrolysin O (ALO), another secreted virulence factor, is a cholesterol-dependent cytolysin that targets host cell membranes. ALO binds to cholesterol, forming large pores in cell membranes, which disrupts membrane integrity and leads to cell lysis. Strains secreting ALO cross host cell layers more effectively than ALO-deficient strains, highlighting its role in virulence. ALO primarily targets immune cells, including monocytes, neutrophils and macrophages, promoting the bacterium’s evasion of the host’s initial immune response and facilitating systemic spread [50,60].

The poly-D-glutamic acid capsule of *B. anthracis* serves as a protective shield against immune attack. Weakly immunogenic, the capsule prevents opsonization, which is the immune system’s method of marking pathogens for destruction. This allows the bacteria to evade phagocytosis and continue its proliferation within the host. The capsule’s production is controlled by the pXO2 plasmid and is upregulated in response to elevated carbon dioxide levels, signaling the bacterium’s transition from the environment to a host [50,61].

**Table 1:** Virulence factors of *B. anthracis* and their roles in anthrax pathogenesis.

Virulence Factor (Encoded by)	Description	Function	References
Protective Antigen (PA) (pXO1 plasmid)	83 kDa protein serving as a central component of anthrax toxins. Binds to receptors CMG2 and TEM8 via MIDAS (metal–ion dependent adhesion site). Cleaved by furin proteases into PA20 (removed) and PA63 (active). Forms octameric rings, binding LF or EF.	Facilitates entry of LF and EF into host cells. Forms a channel in the endosome membrane for LF/EF translocation.	[57]
Lethal Factor (LF) (pXO1 plasmid)	Zn-dependent protease targeting MAPKKs. Requires PA to enter host cells.	Inactivates MAPK signaling, disrupting intracellular signaling. Hampers host immune response and contributes to immune evasion.	[52,53]
Edema Factor (EF) (pXO1 plasmid)	Adenylate cyclase enzyme. Requires PA to enter host cells.	Increases intracellular cAMP levels. Disrupts cellular homeostasis, impairs immune cell functions and contributes to edema formation.	[55,56]
Antiphagocytic Capsule (pXO2 plasmid)	Composed of poly-D-glutamic acid.	Prevents recognition and phagocytosis by immune cells, allowing bacterial survival and systemic spread.	[61]
Anthrolysin O (Chromosomally encoded)	Cholesterol-dependent cytolysin	Forms large pores in cell membranes, disrupts membrane integrity leading to cell lysis. Targets immune cells, promotes bacterium’s evasion of the host’s initial immune response and facilitates systemic spread	[50,60]

### 4.1. Anthrax Pathogenesis

LF is a mettaloproteinase that cleaves mitogen-activated protein kinase kinases (MEKs), which causes inactivation of the three key mitogen-activated protein kinase (MAPK) pathways. These pathways act in response to several stimuli, such as proinflammatory cytokines, heat shock and many other cellular stresses, which play critical roles in cell survival [62]. Macrophages from some strains of rat and mouse, such as Fischer rats and BALBc mice, are sensitive to lethal toxin (LT), whereas a few strains, such as those from C57BL6 mice and Lewis rats, are resistant [63]. It was shown that sensitivity to lethal toxin is due to polymorphisms in a single gene, *Nlrp1* in rats and *Nlrp1b* in mice [64], and not due to cleavage of MEKs. These genes encode NOD-like receptor protein NLRP1, a component of NLRP1 inflammasome (multi-protein complex), which activates caspase-1. Lethal toxin can cleave NLRP1/NLRP1b from sensitive mice/rats, causing inactivation of NLRP1 inflammasome [52] and leading to caspase-1-mediated death of macrophages, called pyroptosis. Later, an interesting observation was reported, that while LT sensitivity of rat is directly related to NLRP1 [64], mice are killed in a manner independent of their macrophage sensitivity to the toxin. This observation suggests the role of other cell types in lethality [65].

The edema factor of Edema Toxin (ET) has adenylyl cyclase activity, which increases cellular cAMP levels, causing multiple effects such as skin and liver edema, and it can induce fluid influx in the intestinal lumen, causing extensive tissue damage and death [66]. cAMP has been shown to have at least two major targets, which include protein kinase A (PKA) and an exchange protein activated by cAMP. Binding of cAMP to regulatory units of PKA causes the release of a catalytic monomer which is activated by ATP, leading to phosphorylation of many substrates. Increased levels of cAMP by cholera toxins is known to cause intestinal fluid secretion by activating cystic fibrosis transmembrane conductance regulator [67], and possibly this mechanism is also followed by ET-induced intestinal secretion.

In the beginning of infection, anthrax toxin complex (LT and ET) weakens the immune system, which helps in the establishment of the disease. In later stages, toxins, together with bacterial infection, cause death by targeting the liver and other vital organs of the host [66].

### 4.2. Anthrax Toxin in Cancer Therapeutics

Cancer is a leading cause of death worldwide and was responsible for approximately 9.6 million deaths in 2018, as per a WHO report [68]. Due to the growing global cancer burden [69], there is an urgent need to develop safe, effective and accessible therapies to treat this slow poison.

One of the primary front-line therapies for cancer is the surgical removal of the tumor from a patient’s body. Another mainstay treatment is radiation therapy, in which ionising radiation is used to irradiate the cancerous region that leads to tumor death due to DNA damage. The mechanism of this therapy is generic and non-selective; thus normal, surrounding tissue is also affected that can lead to acute and/or late toxicity [70]. Chemotherapy, another common method of treatment, works on the principle of utilising toxic compounds and drugs to eliminate cancerous cells [71,72]. Over the years, various natural medicinal compounds and anti-cancer drugs have been devised to target tumors more quickly and effectively while minimizing damage to other surrounding healthy cells [72,73,74,75]. Different types of chemotherapy affect target cells in various ways. Certain treatments may directly modify the quality of cellular proteins, disrupting their functionality and interfering with essential major cellular physiological processes while other treatments target crucial cellular enzymes or may modify cellular metabolism. Chemotherapy also interferes with some critical cellular processes, such as, drug resistance, programmed cell death/apoptosis, DNA damage and replication or immune reactions [76,77,78,79,80]. Resistance to different chemotherapeutic agents may also develop [71].

The conventional therapies to treat cancer as discussed above have limitations and side-effects beyond imagination. Thus, in the pursuit of scrupulous and precise methods, researchers explored the molecular-targeted therapies which reduced poor drug accumulation in the tumor cell and increased efficiency [81]. Over the past decades, bacteria-mediated cancer therapy has emerged to be one of the novel therapies to overcome the limitations of the conventional therapies [49]. To date, three recombinant immunotoxins have received marketing approval for the treatment of hematological cancers namely moxetumomab, pasudotox (anti-CD22) and Denileukin diftitox (Ontak^®^) [82]. Bacteria are known to have anti-tumour property by triggering the immune response through various mechanisms [82]. Bacterial toxins or AB toxins have been efficiently used to transport toxic subunits into the cell [81]. The AB toxins are classified into different groups based on their structure (Table 2) [81].

**Figure 7:**
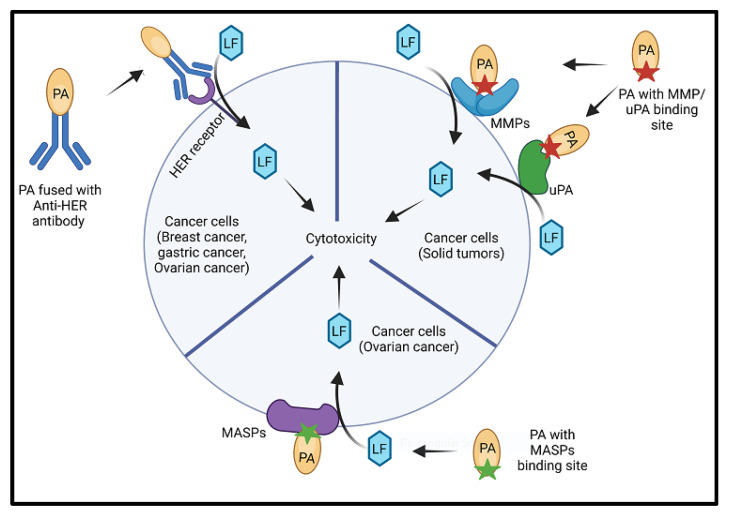
Schematic representation of role of anthrax toxin in cancer treatment.

Both Tumor endothelial marker-8 (TEM8 or ANTRX1) and capillary morphogenesis gene-2 (CMG2 or ANTXR2) play a role in tumor angiogenesis, making them potential targets for cancer therapy [83,84,85,86,87]. This possibility is aided by the presence of unique features in the anthrax toxin proteins that allow their engineering for conversion into specific anticancer agents [88]. For this purpose, their specificity towards tumor cells must be increased. One way of achieving this was based on the prerequisite of proteolytic activation of PA on the cell surface along with the understanding that tumor cells produces higher level of urokinase-type plasminogen activator (uPA) and cell-surface matrix metalloproteinases (MMPs) (Figure 7). Hence, the RKKR sequence, which is the furin target sequence on PA, was replaced with sequences recognized by MMP or uPA, creating targeted anti-tumor agents. [89,90,91,92]. The specificity for tumors was further increased by designing intermolecular complementing PA variants with the presence of both MMPs and uPA for activation [92,93,94]. This method, based on the observation that tumor and stromal cells in cancer often overexpress MMPs and uPA [95,96,97], ensures that these PA variants are selectively activated in solid tumours. This selective activation facilitates the targeted delivery of effector proteins, e.g., LF or recombinant LF fusion proteins, into the cytoplasm of tumour target cells where they can produce various cytotoxic effects.

The intrinsic activity of native LF involves inactivation of mitogen- activated protein kinase kinases (MEKs), which shuts down the RAS-RAF-MEK-ERK (Rat Sarcoma—Rapidly Accelerated Fibrosarcoma—Mitogen-Activated Protein Kinase Kinase—Extracellular Signal-Regulated Kinase) signalling pathway [98,99]. Interestingly, oncogenic mutations in this pathway are commonly observed in human cancers [100]. Therefore, the inherent activity of LF against this pathway highlights another distinct characteristic of the engineered anthrax lethal toxins that enhances their efficacy in tumor targeting. These attributes have positioned these engineered LF toxins as a promising new category of powerful agents for targeted cancer treatment.

The toxin is re-engineered and modified such that it is specific and toxic for cancer cells. In one such study, the anthrax toxin was fused with zymogen activating prodrug and to make it specific for ovarian cancer cells, the PA was modified such that now it could be activated my membrane-anchored serine proteases (MASPs) instead of the classical activation by furin cleavage at RKKR because the MASPs are overexpressed in the ovarian cancer. Upon activation the LF factor is translocated to cytosol where it inactivates MAP kinase pathways thus modulating gene expression [101]. Breast cancer is one of the most diagnosed cancers in women. These are heterogeneous tumours, which exhibit over-expression of the MAPK/ERK pathway, which is involved in the regulation of cell proliferation, differentiation and death. LF upon translocation to cytosol inhibits MAPK/ERK pathway due to zinc- activated metalloprotease and thus inhibits the invasion and migratory capacities of breast cancer cells. ERK inhibits cell adhesion in order to promote cell migration by inactivating RhoA and LeTx treatment increases this activity in addition to the Cdc42 [102].

The PA domain can be mutated to target HER2 receptor, which is commonly found overexpressed in the breast, gastric and ovarian carcinomas [103,104,105,106,107]. They fused a high-affinity Affibody specific for the HER2 receptor namely ZHER2:342 to the C-terminus of the PA domain of the anthrax toxin. This antibody was derived from the z-domain of the *Staphylococcus aureus* protein A. These fusion proteins exhibited a cytocidal effect on the HER2 positive tumour cells [103,108].

## 5. Role of Kinases and Phosphatases in Sporulation, Germination and Virulence of *Bacillus anthracis*

Protein phosphorylation plays a pivotal role in regulating bacterial pathogenesis, a concept that traces its origins to the groundbreaking discovery by Edmond Fischer and Edwin Krebs in 1955 [109]. This dynamic regulatory mechanism involves the addition and removal of phosphate groups on specific amino acids, a process catalyzed by protein kinases and phosphatases, which alter the activity and function of target proteins [3,110]. In *B. anthracis*, the role of protein phosphorylation is especially significant orchestrating essential stages of its life cycle, including toxin production and virulence [4]. Central to this regulation is AtxA, the master virulence regulator that controls the expression of anthrax toxin and capsule biosynthesis genes [4,111,112,113]. AtxA contains two phosphotransferase system regulation domains (PRDs) and an EIIB-like domain, which can be phosphorylated at specific histidine residues. Phosphorylation at His199 in PRD1 is essential for AtxA’s DNA-binding ability, while dephosphorylation at His379 in PRD2 and the EIIB domain promotes AtxA dimerization, the active form required for its regulatory function [113,114,115]. This regulation is mediated by the phosphoenolpyruvate phosphotransferase system (PEP-PTS), a multicomponent system that serves as a key link between carbon source availability and the regulation of virulence factors [116,117]. In *B. anthracis*, deletion of PTS proteins (HPr and Enzyme I) results in decreased *atxA* transcription and anthrax toxin production indicating the crucial role of PTS in anthrax virulence [118]. By influencing the expression of genes involved in toxin production, this system helps *B. anthracis* adjust their pathogenic potential according to environmental nutrient conditions. AtxA’s role extends beyond toxin production; it also influences the bacterium’s decision to sporulate or maintain a virulent state. High levels of AtxA are linked to increased toxin expression and reduced sporulation, a strategic balance that allows *B. anthracis* to either persist in the environment as spores or actively infect and replicate within a host [111]. This dual role of AtxA highlights the sophisticated regulatory mechanisms driven by phosphorylation that enable *B. anthracis* to switch between survival modes based on nutrient availability and other host-derived signals. In addition to histidine phosphorylation, serine/threonine kinases and phosphatases also play crucial roles in the virulence pathways of *B. anthracis*. Strains lacking the ser/thr kinase (PrkC) and phosphatase (PrpC) pair exhibit attenuated virulence and impaired bacterial survival in macrophages in animal models of anthrax [119]. The serine/threonine phosphatase PrpN is also vital for regulating anthrax toxin synthesis. Mutant strains deficient in PrpN show reduced expression of anthrax toxins (PA and LF) and the toxin activator protein AtxA [16]. This defect is linked to the phosphorylation of CodY at Ser215 in its DNA-binding domain, which prevents its binding to the *atxA* promoter, a critical step in activating anthrax toxin gene expression [16,120,121]. 

In terms of phosphorylation mediated signaling pathways in bacteria, the most well-studied signaling mechanisms is a two-component systems (TCSs) [122,123,124], with one component is a sensor histidine kinase (HK), which is a membrane bound and the other is a response regulator (RR), which is a cytoplasmic. On stimulation, the HK component autophosphorylates on a conserved histidine residue and the phosphate group is then transferred to the response regulator at its specific aspartic acid residue, thereby activating the RR. This RR then binds to the target DNA and alters the gene expression [125,126,127]. 

In *B. anthracis*, TCSs play a vital role in modulating virulence factors, including the lethal and edema toxins. For instance, the BrrA-BrrB TCS regulates the expression of anthrax toxin genes during infection. Disruption of either component in this system significantly reduces the bacterium’s ability to produce toxins, resulting in attenuated virulence [128]. Moreover, TCSs, such as the HssRS and HitRS systems, allow *B. anthracis* to sense and respond to heme toxicity and cell envelope stress, respectively [127,128,129] *B. anthracis* uses the HssRS two-component system to sense and respond to heme toxicity during infection. In the host, HssRS regulates the expression of the heme transporter, HrtAB (heme detoxification system) and allows *B. anthracis* to proliferate to high levels in vertebrate tissues during anthrax pathogenesis by acquiring iron from host heme while avoiding heme toxicity [129]. Interestingly, HitRS triggers the expression of the same gene pool as the heme sensing HssRS TCS [127,130].

Phosphorelays, which are more elaborate versions of two-component systems, enable more complex regulatory control in *B. anthracis*. These systems involve multiple phosphotransfer steps, allowing for fine-tuned regulation of sporulation and other cellular processes. Spo0A, the master regulator of sporulation is activated by a complex phosphorelay system involving multiple histidine sensor kinases (HKs) and response regulators (RRs) [131,132]. The main proteins involved are Spo0F (response regulator), Spo0B (phosphotransferase) and Spo0A (master transcription factor) [13,132,133]. The goal of the phosphorelay is to activate Spo0A through phosphorylation (Spo0A~P) that functions as a transcription factor and regulates genes critical for sporulation and virulence. This activation is reversed by the Spo0E and Rap phosphatase, which dephosphorylates Spo0A and Spo0F RR, providing an additional layer of control [13,134]. Additionally, two other TCSs, BAS1213–1214 and BAS0540–0541, have been identified in sporulation modulation. Upregulation of either BAS1213 or BAS0540 RRs significantly reduces sporulation efficiency [135,136]. Serine/threonine phosphorylation also impacts sporulation, as strains deficient in the serine/threonine phosphatase PrpN show marked sporulation defects [16]. 

Beyond its role in toxin production and sporulation, protein phosphorylation in *B. anthracis* regulates a range of virulence-associated traits, including spore germination, chaining morphology and biofilm formation. The serine/threonine kinase PrkC is particularly significant in regulating these lifecycle transitions. PrkC phosphorylates key enzymes such as enolase and GroEL, thereby modulating spore germination and biofilm development [137,138]. Enolase overexpression during sporulation disrupts germination and PrkC-mediated phosphorylation of enolase reduces its catalytic activity while also modulating its expression and localization. This regulatory phosphorylation by PrkC, therefore, plays a crucial role in controlling the overall spore germination process [138]. The PrkC-PrpC kinase-phosphatase pair also reversibly controls the phosphorylation of GroEL, an essential chaperone involved in biofilm formation [139]. Additionally, PrkC also regulates chain formation, a virulence factor that aids in immune evasion, as strains deficient in PrkC show severe defects in chain formation [140]. 

In conclusion, protein phosphorylation serves as a central regulatory mechanism that allows *B. anthracis* to fine-tune its response to environmental signals, optimize energy usage and coordinate virulence factor expression. A deeper understanding of these phosphorylation driven pathways will provide valuable insights into the molecular strategies employed by *B. anthracis* to establish infection and evade host defences. This knowledge opens new opportunities for targeted therapeutic interventions. Drugs designed to modulate specific phosphorylation events could effectively disrupt key processes such as virulence factor expression, sporulation and biofilm formation; thereby weakening the pathogen’s ability to adapt and persist within the host. Such an approach offers the potential to attenuate infection and reduce the severity of anthrax cases. As research in this field advances, bacterial kinases and their associated phosphorylation events are emerging as attractive targets for drug developments [141,142]. Moreover the broader implications of targeting phosphorylation in bacterial pathogens extend beyond anthrax. The conserved nature of kinase-regulatory pathways across many bacterial species suggest that insights gained from studying *B. anthracis* could inform development of therapies for other bacterial infections. The intricate interplay between phosphorylation-driven processes and virulence underscores the importance of continued research into these regulatory mechanisms.

## 6. Prevention and Treatment of Anthrax

Vegetative forms of *B*. *anthracis* are susceptible to most antibiotics with penicillin G and amoxicillin or ciprofloxacin [42]. In inhalation anthrax, mechanical ventilation can be given to patients to prevent respiratory failure. For treatment of gastrointestinal anthrax, combination of penicillin G with streptomycin or aminoglycosides in adequate dose intravenously could be effective. Sometimes, surgical treatment to treat gastrointestinal anthrax may be required. Although rare, anthrax meningoencephalitis requires antibiotic treatment for a longer time. It has been reported that some strains of *B. anthracis* are naturally resistant to penicillin and in such cases appropriate antibiotics need to be administered to the patients. Often anthrax infections are diagnosed late and by this time its toxin levels become lethal. While antibiotics can kill bacteria, it can neutralize anthrax toxin. Two monoclonal antibodies, namely Raxibacumab [143] and obiltoxaximab [144], which targets protective antigens have been developed and approved by Food and Drug Administration USA (US FDA) for treatment of patients.

Vaccines have been one of the most effective public health tools, drastically reducing the prevalence of many infectious diseases. In the case of anthrax, several types of vaccines have been developed to target its unique mechanisms of infection [145]. Whole-cell vaccines use live, attenuated (weakened) forms of *B. anthracis*, allowing the immune system to build a strong defense. However, they require multiple doses and sometimes cause side effects. Protein-based vaccines, on the other hand, focus on specific anthrax proteins, like the protective antigen (PA), which is crucial for toxin entry into host cells. PA-based vaccines are safer and stimulate immunity against anthrax toxins without using live bacteria [146,147]. Another category of vaccines are capsule vaccines that utilize the capsule composed of poly-γ-D-glutamic acid to target the capsule that surrounds *B. anthracis*. Toxin vaccines, on the other hand, use anthrax toxins such as protective antigen (PA), lethal factor (LF) and edema factor (EF) in combination to broaden immunity and neutralize the toxins effectively.

Historically, there are three major vaccines in use for anthrax, namely, BioThrax (AVA or Anthrax vaccine adsorbed), Anthrax vaccine precipitated (AVP) and Live anthrax vaccine (LAV). BioThrax and AVP are cell-free protein-based vaccines, whereas LAV is a spore-based vaccine (Table 3). Recently, the US FDA has approved the use of another anthrax vaccine named CYFENDUS™ (AV7909), which is similar to BioThrax but contains an additional adjuvant, CPG 7909, which enhances its immunogenicity.

BioThrax (AVA), developed in the United States, is derived from a cell-free filtrate of the *B. anthracis* strain V770-NP1-R (BioThrax | FDA) [148,149]. It contains the PA protein, which stimulates immunity against anthrax toxins. BioThrax is administered intramuscularly in a multi-dose series followed by booster doses to maintain immunity. Anthrax Vaccine Precipitated (AVP), used in the UK is a cell-free formulation made from the Sterne 34F2 strain of *B. anthracis* [146,150]. It includes PA, LF and small amounts of EF protein, generating a broader immune response than PA alone. AVP also requires multiple doses with boosters for long-term protection. Live Anthrax Vaccine (LAV) developed in Russia, was composed of non-encapsulated live spores from two different strains of *B. anthracis* (STI-1 and NO 3) [151,152]. In the next version of this vaccine, LAV STI-1 only STI-1 strain was used. LAV can be administered cutaneously or subcutaneously and requires multiple annual doses to sustain immunity.

Subsequently, various studies have been conducted to increase the immunogenicity of the above vaccines by utilising different adjuvants [153]. For example, AV7909 is AVA adjuvanted with CPG 7909, a immunostimulatory oligodeoxynucleotide [154,155]. Similarly, STI-1+ PA is LAV adjuvanted with PA adsorbed on aluminium hydroxide [152].

**Table 3:** Major anthrax vaccines in use and their properties.

Vaccine Name	Vaccine Type	Details (References)
BioThrax^®^ (AVA or Anthrax vaccine adsorbed)	Derived from cell-free extracts of an avirulent strain of *B. anthracis* (V770-NP1-R strain) with alum as the adjuvant. Immunogen- PA	Pre-exposure prophylaxis—intramuscular injection. Post-exposure prophylaxis—subcutaneously. Pre-exposure prophylaxis—Primary doses at 0, 1 and 6 months. After completion of primary series, booster vaccinations at 6 and 12 months followed by annual booster thereafter. Post-exposure prophylaxis—Doses at 0, 2 and 4 weeks. [148,149,156]
CYFENDUS™, also known as AV7909	Same as BioThrax with an additional adjuvant, CPG 7909 in addition to alum.	Administered by intramuscular injections and only during post-exposure prophylaxis. Two doses at Week 0 and Week 2. [157]
AVP or Anthrax vaccine precipitated	Derived from cell-free extract of the vaccine strain *B. anthracis* Sterne 34F2 strain, precipitated with alum. Immunogen- Composed mostly of PA and LF. Also, traces of EF present.	Intramuscular injection. Doses at 0, 3, 6 and 32 weeks. Boosters annually thereafter [146,150]
Live anthrax vaccine (LAV).	Live-attenuated anthrax vaccine Consists of dry spores of nonencapsulated *B. anthracis* variants (STI-1 and NO 3)	Cutaneous and subcutaneous administration. Annual doses for three years, with boosts every two years thereafter. [151,152]

Additionally, recombinant vaccines and DNA vaccines have also been developed. For instance, plasmid DNA vaccines coding for PA and LF [158] and adenovirus packaged vaccine [159,160,161]. A live attenuated vaccine with mutations in genes that make the bacillus acapsular has also been developed [162]. Also, a whole-cell vaccine consisting of killed but metabolically active bacteria is being used as a vaccine [163].

Anthrax infections in humans and animals are often diagnosed late and can be fatal [164,165] However, early diagnosis allows effective treatment with antibiotics, immunotherapy and toxin inhibitors. The primary treatment involves antibiotics such as ciprofloxacin, levofloxacin, penicillin and doxycycline, often combined with antitoxins [166]. Ciprofloxacin is a commonly used antibiotic to treat anthrax globally [167].

Extensive research has been conducted to develop specific inhibitors targeting virulence factors like LF, EF, PA and capsule. Efforts also focus on creating inhibitors for anthrax toxin receptors and furin endoproteases necessary for toxin activation. Recently, the antimalarial drug amodiaquine (AQ) has been suggested as adjunctive therapy for anthrax, as it inhibits anthrax toxin endocytosis in the host [168]. The FDA currently recommends three antitoxins for systemic anthrax cases: Raxibacumab, ANTHRASIL (Anthrax Immunoglobulin Intravenous—AIGIV) and Obiltoxaximab (Anthim/ETI-204) [169,170,171]. These antitoxins block the binding of PA to cell surface receptors, preventing the translocation of LF and EF.

Research into small-molecule inhibitors for *B. anthracis* virulence factors, especially lethal factors, has progressed significantly. Hydroxamate-based drugs, benzylamine-based drugs and auranofin have shown efficacy against LF activity in vivo [172,173,174]. Combining ciprofloxacin with potent LF inhibitors offers promising future treatment options for anthrax.

Despite progress, current anthrax treatments have limitations, such as incomplete neutralization, residual toxicity, short-term protection and ineffectiveness against meningitis due to the inability to cross the blood-brain barrier. Thus, new vaccines and drugs are needed to address these issues and enhance anthrax prophylaxis strategies.

## 7. Conclusions and Future Direction

Anthrax, caused by *Bacillus anthracis*, presents a unique challenge in diagnosis and treatment due to its initial flu-like symptoms. This similarity to common illnesses often leads to delayed diagnosis, allowing the infection to progress to potentially fatal levels. As the disease progresses undetected, toxin levels in the body can reach dangerous thresholds, significantly increasing the risk of mortality. In cases of advanced infection, a multi-faceted approach combining antibiotics with toxin-neutralizing agents has shown promise, provided they don’t cause adverse reactions Over the past two decades several small molecules like toxin inhibitors and antibiotics, reagents and antibodies have been developed that can help in the treatment of anthrax. A comparative efficacy study of these therapeutic agents is crucial for improving our readiness to tackle anthrax outbreaks. Vaccination remains another important aspect of anthrax prevention. Currently, the standard vaccine, consisting of culture supernatant proteins adsorbed on alum, is primarily administered to occupationally exposed individuals (such as farmers, zoo employees and wool and hide handlers) and military personnel. However, this vaccine is based on older technology and may benefit from modern technology. The rarity of anthrax in the general population presents a challenge for vaccine and treatment development, as pharmaceutical companies have little financial incentive to invest in these areas. Nevertheless, given the potential for drug resistance, it would be prudent to develop effective vaccines for all infectious diseases, including anthrax, before a crisis emerges. A surge in funding during the early 21st century significantly advanced our understanding of anthrax pathogenesis and its lethal mechanisms. However, global funding for anthrax research has since declined, hampering the testing of potential protective molecules and the development of recombinant vaccines. Recent studies demonstrating the protective properties of the anthrax capsule are promising. A recombinant vaccine incorporating various virulence factors, including the capsule, could prove valuable for at-risk populations and future preparedness efforts. This approach could lead to more effective and broadly applicable vaccines, enhancing our ability to combat potential anthrax threats.

## Figures and Tables

**Table 2:** Types of AB toxin.

AB Toxin	Receptor	Target	Activity	Type	Disulfide Bond Reduction
Diphtheria Toxin (Dtx)	ProHB-EGF	Elongation Factor 2 (EF-2)	ADP-ribosyl transferase	AB (1:1)	NO
Anthrax Toxin (Atx)	TEM8, CMG2	Protein Kinases MAPKK	EF = Adenylate Cyclase LF = Zn metalloprotease	AB_7_	NO
Shiga Toxin (Stx)	Gb3 glycolipid	rRna (28S)	RNA N- glycosidase	AB_5_	YES
Cholera Toxin (Ctx)	GM1 ganglioside	Adenylate cyclase	ADP-ribosyl transferase	AB_5_	YES
Pseudomonas aeruginosa- exotoxin A	CD91 (a2- macroglobulin receptor)	ADP ribosylation of elongation factor 2 (EF 2)	Protein synthesis Inhibition	AB	NO

## Data Availability

No new data were created or analyzed in this study.
